# *In vitro* and *in vivo* Biocompatibility of Alginate Dialdehyde/Gelatin Hydrogels with and without Nanoscaled Bioactive Glass for Bone Tissue Engineering Applications

**DOI:** 10.3390/ma7031957

**Published:** 2014-03-06

**Authors:** Ulrike Rottensteiner, Bapi Sarker, Dominik Heusinger, Diana Dafinova, Subha N. Rath, Justus P. Beier, Ulrich Kneser, Raymund E. Horch, Rainer Detsch, Aldo R. Boccaccini, Andreas Arkudas

**Affiliations:** 1 Department of Plastic and Hand Surgery, University Hospital of Erlangen, Friedrich Alexander University Erlangen-Nuremberg, 91054 Erlangen, Germany; E-Mails: Ulrike.Rottensteiner@uk-erlangen.de (U.R.); diana-dafinova@hotmail.de (D.D.); subharath@iith.ac.in (S.N.R.); Justus.Beier@uk-erlangen.de (J.P.B.); ulrich.kneser@bgu-ludwigshafen.de (U.K.); Raymund.Horch@uk-erlangen.de (R.E.H.); 2 Institute of Biomaterials, Department of Materials Science and Engineering, Friedrich Alexander University Erlangen-Nuremberg, 91058 Erlangen, Germany; E-Mails: bapi.sarker@ww.uni-erlangen.de (B.S.); domiheusinger@web.de (D.H.); rainer.detsch@ww.uni-erlangen.de (R.D.); 3 Department of Hand, Plastic and Reconstructive Surgery-Burns Centre, BG Trauma Centre Ludwigshafen and Department of Plastic Surgery, University of Heidelberg, 67071 Ludwigshafen, Germany; 4 Department of Biomedical Engineering, Indian Institute of Technology, Hyderabad 502205, India

**Keywords:** alginate dialdehyde, gelatin, nano-Bioglass^®^, tissue engineering, biocompatibility, mesenchymal stem cells

## Abstract

In addition to good mechanical properties needed for three-dimensional tissue engineering, the combination of alginate dialdehyde, gelatin and nano-scaled bioactive glass (45S5) is supposed to combine excellent cellular adhesion, proliferation and differentiation properties, good biocompatibility and predictable degradation rates. The goal of this study was to evaluate the*in vitro* and *in vivo* biocompatibility as a first step on the way to its use as a scaffold in bone tissue engineering. *In vitro* evaluation showed good cell adherence and proliferation of bone marrow derived mesenchymal stem cells seeded on covalently crosslinked alginate dialdehyde-gelatin (ADA-GEL) hydrogel films with and without 0.1% nano-Bioglass^®^(nBG). Lactate dehydrogenase (LDH)- and mitochondrial activity significantly increased in both ADA-GEL and ADA-GEL-nBG groups compared to alginate. However, addition of 0.1% nBG seemed to have slight cytotoxic effect compared to ADA-GEL. *In vivo* implantation did not produce a significant inflammatory reaction, and ongoing degradation could be seen after four weeks. Ongoing vascularization was detected after four weeks. The good biocompatibility encourages future studies using ADA-GEL and nBG for bone tissue engineering application.

## 1. Introduction

Alginate is a polysaccharide found in brown seaweeds (Laminaria sp., Macrocystis sp., Lessonia sp. and others). The ability to form hydrogels in the presence of calcium ions that can be easily modulated into various shapes, the good*in vitro* and *in vivo* biocompatibility, low toxicity and its low price make alginate apromising candidate for tissue engineering applications[1–4]. However, alginate shows a low viscosity, making precise 3D printing difficult [2]. Furthermore, it does not degrade, but rather dissolves in surrounding physiological media of mammals and remains in the body [5]. Another limitation is that it does not promote cell interactions[6], which is a crucial feature in tissue engineering.

One possibility for overcoming these limitations is covalent crosslinking of alginate and gelatin. Gelatin is obtained by acidic or basic hydrolysis of collagen, a substance found in the extracellular matrix (ECM). Its use has been shown to promote cell adhesion and proliferation*in vitro* [7,8].Furthermore increase in gelatin content results in a higher viscosity of the hydrogel thus making it more suitable for three-dimensional tissue engineering applications[2].

Both alginate and gelatin have shown good biocompatibility in numerous *in vivo* studies with and without encapsulated cells [4,9–11]. Gelatin has been shown to promote angiogenesis [10], an important feature in tissue engineering, where survival of cells in large implants depends on formation of new blood vessels. In addition good osteogenesis has been observed when using alginate/gelatin scaffolds in bone tissue engineering [11].

Bioactive glasses are increasingly considered in bone tissue engineering due to their angiogenic[12]and osteogenic[13] properties. Recently, nano-scaled bioactive glass particles (nBG) have been integrated into various biopolymers, such as alginate[14], gelatin[15], collagen[16] and chitosan[17–19]. Stimulation of osteogenic cell proliferation and differentiation[20] and formation of bone-like apatite clusters[19] have been observed. Due to their higher specific surface, nano-scaledparticles are likely to show faster solubility, higher protein adsorption and therefore higher bioactivity than larger particles[21].

To the best of our knowledge, this is the first study evaluating the biocompatibility of covalently crosslinked alginate dialdehyde-gelatin hydrogel with and without nBG*in vitro* and *in vivo*, as well as degradation and vascularization behavior of these materials after subcutaneous implantation in rats.

## 2. Methods

### 2.1. In vitro: Preparation of Hydrogels

#### 2.1.1. Materials

Sodium alginate (derived from brown algae, suitable for immobilization of micro-organisms, guluronic acid content 65%–70%), gelatin (Bloom300, Type A, porcine skin, suitable for cell culture) were purchased from Sigma-Aldrich (Munich, Germany). Sodium metaperiodate and calcium chloride di-hydrate (CaCl_2_2H_2_O) were purchased from VWR international (Leuven, Belgium). 45S5 nBG was synthesized by flame pyrolysis [21] and received from ETH (Zurich, Switzerland). The composition of the nanosized bioactive glass is 47.8 wt% SiO_2_, 25.1 wt% CaO, 22.6 wt% Na_2_O, and 4.6 wt% P_2_O_5_. The particle size of nBG is in the range of 20–60 nm [22].

#### 2.1.2. Synthesis of Alginate Dialdehyde-Gelatin Crosslinked (ADA-GEL) and Alginate Hydrogels

The synthesis process of ADA-GEL hydrogel was elaborately described in our previous study [23]. Shortly, ADA-GEL hydrogel was synthesized by covalent crosslinking of alginate dialdehyde (ADA) and gelatin. ADA was synthesized by controlled oxidation of sodium alginate with sodium metaperiodate (oxidizing agent) in equal volume of ethanol-water mixture. After 6 h the reaction was quenched by adding ethylene glycol (VWR int., Leuven, Belgium) and dialyzed against ultrapure water (Direct-Q^®^, Merck Millipore, Darmstadt, Germany) using a dialysis membrane (MWCO: 6000–8000 Da, Spectrum Lab, Rancho Dominguez, CA,USA) for 7 days and then lyophilized. Gelatin solution (5% w/v, in water) was added slowly in ADA solution (5% w/v, in phosphate buffered saline (PBS)) under continuous stirring to facilitate crosslinking between ADA and gelatin.

To prepare alginate solution, sodium alginate 2% (w/v) was dissolved in PBS and the prepared solution was sterilized by filtration using 0.45 μm filter.

#### 2.1.3. Preparation of Films

ADA and gelatin solution were sterilized by filtration through 0.45μm and 0.22 μm filter (Carl Roth GmbH + Co. KG, Karlsruhe, Germany), respectively, prior to mixing. Sterilized ADA and gelatin solutions were mixed in equal volume ratio. nBG particles were sterilized by dry heat sterilization technique. Sterilized nBG particles were added to ADA solution prior to mixing with gelatin to prepare ADA-GEL-nBG hydrogels that contained 0.1% (w/v) nBG.

Alginate, ADA-GEL and ADA-GEL-nBG hydrogels were casted in sterile petridishes. Ionic gelation of the casted films was conducted with calcium chloride (CaCl_2_) solution (0.1 M). Serum free Dulbecco’s modified Eagle’s medium (DMEM) (Gibco^®^, Darmstadt, Germany) was used to wash the hydrogel films and cut by punching to make the films of desired size and shape (circular, diameter 13.5 mm and thickness 1.5 mm).

For the*in vitro* study, ADA-GEL and ADA-GEL-nBG hydrogels along with alginate (control hydrogel) was used.

### 2.2. Isolation and Cultivation of MSCs

Bone-marrow derived mesenchymal stem cells (MSCs) were isolated from an adult male Lewis rat. The animal was deeply anesthetized using isoflurane in pure oxygen, and both femora and tibiae were aseptically explanted. Each bone marrow cavity was flushed with 15 mL MACS buffer (PBS, Biochrom + 2% fetal calf serum, Biochrom, Berlin, Germany), and the resulting cell suspension was centrifuged at 1400 rpm for 4 min. The supernatant was discarded and the cell pellet resuspended in culture medium (DMEM + Ham’s F12 1:1, Biochrom, Berlin, Germany; 20% FCS, Biochrom, Berlin, Germany; 1% L-Glutamine, Gibco, Darmstadt, Germany). The cell pellet was filtered through a 100 μm pore size cell strainer (BD, Heidelberg, Germany). Mononuclear cells were isolated using density gradient centrifugation (Histo-Paque 1077, Sigma Aldrich, Munich, Germany). Cells were seeded on a standard plastic 24 well plate (Greiner Bio-One, Frickenhausen, Germany) with 2 × 10^6^ cells/well. After 36 h, the non-adherent cells were removed by washing each well with PBS, and fresh medium was supplied.

Cells were monitored daily, and medium was changed every 2–3 days; passaging was done at 70%–80% confluency (after 5–6 days). Cells were detached using trypsin/EDTA solution (Biochrom, Berlin, Germany) for 4 min, and seeded at 5000 cells/cm^2^ (P1, P2) or 3000 cells/cm^2^ (P3 and following passages) onto standard plastic cell culture flasks (Greiner Bio-One, Frickenhausen, Germany).

### 2.3. In Vitro Cell Attachment and Proliferation Studies: 2D

The prepared circular films of alginate, ADA-GEL and ADA-GEL-nBG hydrogels were transferred into a 24-well plates (VWR int., Leuven,Germany). MSCs were seeded at a density of 100,000 cells/film, cultured in Dulbecco’s modified Eagle’s medium (DMEM) (Gibco^®^, Darmstadt, Germany) supplemented with 10% (v/v) fetal calf serum (FCS) (Sigma-Aldrich, Munich,Germany) and 1% (v/v) penicillin-streptomycin (Gibco^®^, Darmstadt, Germany) and incubated in a humidified atmosphere of 95% relative humidity and 5% CO_2_, at 37°C. Culture medium was changed the day after seeding and then every two days.

#### 2.3.1. Mitochondrial Activity

Mitochondrial activity of seeded MSCs on different hydrogel films was assessed through the enzymatic conversion of tetrazolium salt (WST-8 assay kit, Sigma Aldrich, Munich, Germany) after 48 h of cultivation. Culture medium was completely removed from the incubated films and 1%(v/v) WST-8 assay kit containing culture medium was subsequently added and incubated for 2 h. 100μL of supernatant from each samples were transferred into a 96 well-plate and the absorbance at 450nm was measured with a microplate reader (PHOmo, autobio labtec instruments co. Ltd., Zhengzhou,China).

#### 2.3.2. Lactate Dehydrogenase (LDH) Activity

A commercially available LDH-activity quantification kit (TOX7, Sigma-Aldrich, Munich, Germany) was used to quantify the LDH enzyme activity in cell lysate. After 48 h of incubation the hydrogel films were washed with PBS, and lysis buffer was added to the films (1 mL/film) and incubated for 30 min. Lysates were centrifuged at 2000 rpm for 5 min and 140 μL from the supernatant solutions were transferred to 1 mL cuvettes. 60 mL of master-mix (equal amounts of substrate solution, dye solution, and cofactor solution for LDH assay) were added to each cuvette and incubated for 30 min. The reaction was stopped with 300 μL of 1N HCl, and 500μL of water were added. The absorbance of each solution was measured at 490 and 690 nm using a UV-Vis spectrophotometer (Specord 40, Analytik Jena AG, Jena, Germany).

#### 2.3.3. Fluorescence Staining

To reveal the morphology of the adhered cells after 48 h of cultivation, staining was performed with Rhodamine Phalloidin (Invitrogen, Carlsbad, CA,USA), which selectively stains F-actin. Nuclei were stained with green nucleic acid stain, SYTOX^®^ (Invitrogen, Carlsbad, CA, USA). Phalloidin-Sytox stained MSCs on hydrogel films were visualized by fluorescence microscopy (FM) (Axio Scope A.1, Carl Zeiss Microscopy, Jena,Germany).

#### 2.3.4. Cell Counting

The cell number was identified by counting DAPI-stained cell nuclei using ImageJ software (National Institutes of Health, Bethesda, MD, USA; version 1.47v). Four images of 20× magnification from different areas of each film were used to define the cell uniformity of the film. The entire area of image was calculated and the number of cells was presented per unit area of sample.

### 2.4. In vivo: Subcutaneous Implantation of ADA-GEL Microcapsules

#### 2.4.1. Animals

*In vivo* procedures were approved by the government of Middle Franconia and the according Animal Protection Committee (Az 54-2532.1-24/09).

Six adult, healthy male Lewis rats (weight 303–320g) were used. In each animal, two chambers filled with pure ADA-GEL microcapsules and two chambers filled with ADA-GEL + nano-Bioglass^®^ (nBG) microcapsules were implanted. Time until explantation was 1 week (3 animals) and 4 weeks (3 animals). Six chambers per group and per explantation point were evaluated.Animals were housed in single cages (type 3) in a standardized environment (20–22°C, relative humidity 46%–48%, light/dark cyclus of 12 h), and had free access to water and standard chow (Ssniff, Soest, Germany).

#### 2.4.2. *In Vivo* Implantation

**Figure 1.**
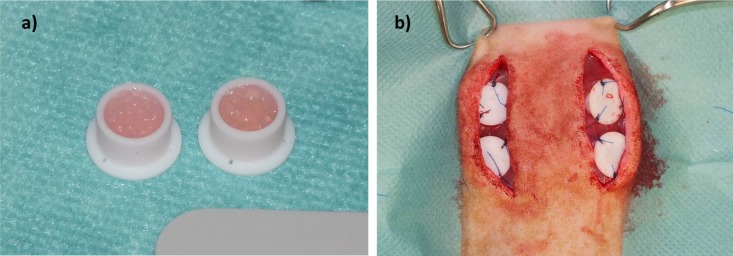
(**a**) Teflon chambers filled with ADA-GEL microcapsules prior to implantation. Inner diameter of chambers = 8 mm; (**b**) Subcutaneous implantation of Teflon chambers.

Microcapsuleswereprepared immediately before subcutaneous implantation.For the *in vivo* study, we selected the materials with best results from*in vitro* analysis (ADA-GEL and ADA-GEL-nBG). Solutions were prepared as described for*in vitro* experiments. Microcapsules from ADA-GEL and ADA-GEL-nBG hydrogels were prepared by pneumatic extrusion technique. Hydrogels were transferred into an extrusion cartridge (Nordson EFD, East Providence, RI, USA), which was connected to a high precision fluid dispenser (Ultimus V, Nordson EFD, East Providence, RI,USA). Microcapsules were generated by applying different air pressure (2.3 bars to 2.5 bars), collected in a beaker containing CaCl_2_ solution (0.1 M) and kept for 10 min to allow for ionic gelation. Subsequently the fabricated microcapsules were sieved and washed three times with medium (DMEM) to remove calcium chloride solution from the surface of the microcapsules.After the crosslinking procedure, microcapsules were transferred into custom made Teflon chambers for subcutaneous implantation (inner diameter 8 mm, height 6 mm, volume approx. 400 μL; Figure 1a). Weight of chambers filled with microcapsules was approximately 300 mg.

Rats were anesthetized using an induction chamber filled with 5% isoflurane in oxygen. After induction, rats were placed on a heating plate and anesthesia was maintained by delivering isoflurane (Isofluran, CP Pharma, Burgdorf, Germany) in oxygen via face mask. Preemptive analgesia was achieved by administration of 0.03 mg/kg buprenorphin (Temgesic, RB Pharmaceuticals, Mannheim,Germany). A single administration of procain penicillin (22000 IU/kg) and dihydrostreptomycin (50 mg/kg) (Veracin compositum, Albrecht, Aulendorf, Germany) was used for prevention of infection.

Rats were placed in sternal recumbency, and the back was clipped and aseptically prepared for surgery. Two paramedian, longitudinal incisions were created on the back, and subcutaneous tissue was removed to expose the underlying muscle. Two chambers were placed on each side with the open side facing downwards, thus bringing the microcapsules in direct contact with the muscle (Figure 1b). Chambers were fixed using USP 4/0 polypropylene(Prolene, Ethicon; Johnson&Johnson, Norderstedt, Germany) sutures, and the skin was closed using USP 4/0 polyglactin 910 (Vicryl Plus, Ethicon; Johnson&Johnson, Norderstedt, Germany). Buprenorphin treatment was continued until the second postoperative day, followed by administration of tramadol (Tramal, 100 mg/mL, Gruenenthal, Aachen,Germany) via drinking water (7.5 mg/100 mL of tap water) for the first postoperative week.

#### 2.4.3. Explantation Procedure

After 1 and 4 weeks, respectively, rats were anesthetized as described above. Animals were placed in dorsal recumbency, and a midline laparotomy was performed. Intestines were exteriorized, and the caudal vena cava and aorta were isolated. A 24 G vein cannula was inserted into the caudal vena cava, the aorta was opened, and the vascular system was flushed with heparinized saline solution. After removing all blood from the vessels, a mixture of India Ink (Lefranc-Bourgeois; Colart Deutschland GmbH, Maintal, Germany; 20 mL), saline solution (0.9%; 20 mL), mannite (Carl Roth GmbH & Co KG, Karlsruhe, Germany; 1.2 g) and gelatin (Carl Roth GmbH & Co KG, Karlsruhe, Germany; 1.5 g) was injected. After cooling at −20°C for 2 h, chambers were explanted and fixed in 4% formaldehyde solution (Histofix 4%, Carl Roth GmbH & Co KG, Karlsruhe, Germany) for 24 h. Microcapsules were carefully removed from the teflon chambers with the underlying muscle and further processed for routine histology.

#### 2.4.4. Histological Evaluation

Three micrometer cross sections were obtained from two standardized planes (1mm left and 1mm right of the central plane) rectangular to the longitudinal axis of the construct. Hematoxylin and eosin (H&E) staining was performed according to standard protocols. Immunohistological stainings were performed for ED1 (detection of macrophages/immune reaction) and Lectin (Bandeiraea Simplicifolia agglutinin, BS-1; endothelial cells).

##### Lectin

Paraffinated sections were dewaxed and cookedin citrate buffer (pH 6) for 1 min at 121°C. After 10 min in 3% H_2_O_2_ (Merck, Darmstadt, Germany), sections were incubated in avidin and biotin blocking solution (Avidin/Biotin Blocking Kit, Vector Labs; Biozol, Eching, Germany). Goat serum (Vector Labs) 10% was applied for 30min. after this, sections were incubated overnight at 4°C in biotinylated Lectin antibody (Isolectin B4 Biotinlabeled, Sigma Aldrich, Munich, Germany; 1:270 in TRIS buffer). The day after, detection was carried out using streptavidin AB Complex/HRP (Dako GmbH, Hamburg, Germany) for 30min, washed, followed by development with DAB+ chromogen (Dako GmbH, Hamburg, Germany) 10 min. Counterstaining was performed with 1:10 Hematoxylin solution.

##### ED1 (CD68)

ED1 is a monoclonal antibody that binds to the rat homologue of human CD68, a 90–110 kDa protein found on macrophages and monocytes [24]. For this staining, sections were dewaxed and cooked at 121°C for 1min. After cooling, a blocking solution (Zytochem Plus AP Polymer Kit, Zytomed Systems, Berlin, Germany) was applied for 5minto minimize background staining. Slides were incubated overnight at 4°C in primary antibody solution (monoclonal mouse anti-rat CD68, Serotec, Düsseldorf, Germany; 1:300). The next day, enhancement reagent (Zytochem Plus AP Polymer Kit) was applied for 30min, followed by an AP coupled secondary antibody (anti-mouse/anti-rabbit, 30min). Development was carried out using Fast Red solution for 20 min, and Hemalaun solution was used for counterstaining.

Images were using an Olympus IX81 microscope (Olympus, Hamburg, Germany) and Digital Camera under 4×, 10× or 20× magnification. Cell proliferation was analyzed by counting of fluorescence stained cell nuclei using a standardized threshold (ImageJ, National Institutes of Health, Bethesda, Md.).

##### Statistics

Statistical analysis of mitochondrial and LDH activity of MSCs and cell number was accomplished by one-way analysis of variance (ANOVA) on the different hydrogel films after 48h of incubation. The pairwise comparison of the means was performed with the Bonferroni’s test (post hoc comparison). P-values < 0.05 were considered statistically significant.

## 3. Results

### 3.1. In vitro Evaluation of Biocompatibility

#### 3.1.1 Cell Viability

**Figure 2.**
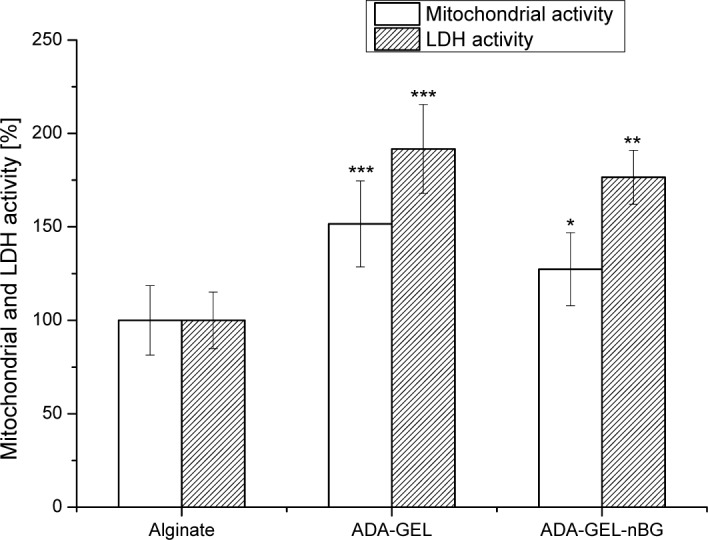
Mitochondrial and LDH activities of rMSCs on alginate, ADA-GEL and ADA-GEL-nBG hydrogel films after 48 h of cultivation. Alginate was used as the control material for each assay. Asterisks denote significant difference compared to alginate hydrogel, *p< 0.05, **p< 0.01 and ***p< 0.001 (Bonferroni’s post-hoc test was used).

The viability of the rat bmMSCs on alginate, alginate dialdehyde-gelatin (ADA-GEL) and alginate dialdehyde-gelatin-nano-Bioglass^®^ (ADA-GEL-nBG) hydrogels after 48 h of incubation was investigated by analyzing the mitochondrial and LDH activities, as presented in Figure 2.

Mitochondrial activity of MSCs for ADA-GEL and ADA-GEL-nBG hydrogels was found to be significantly higher compared to that for the control material, alginate, with 1.5 and 1.3 times increasedfor ADA-GEL and ADA-GEL-nBG, respectively. A similar trend was observed for LDH activity of MSCs, where LDH activity for ADA-GEL and ADA-GEL-nBG was found to be 1.9 and 1.7 folds increased compared to alginate.

#### 3.1.2. Cell Morphology

**Figure 3.**
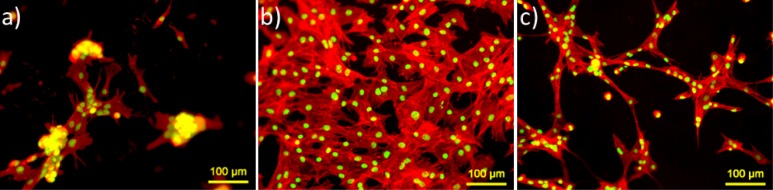
FM images of MSCs adhered on (**a**) alginate; (**b**) ADA-GEL; and (**c**) ADA-GEL-nBG hydrogel films after 48 h of cultivation. The cells were stained for actin (red) and nucleus (green).

Actin cytoskeleton staining of MSCs was performed to visualize overall prospective of cell morphology and cell spreading on the hydrogels (see Figure 3). Ahigh number of cells on ADA-GEL and ADA-GEL-nBG hydrogels was evident, compared to alginate. On alginate films, cells were agglomerated and formed clusters after 48 h.On ADA-GEL, cells were spread in flat shape and interacted with each other in high contact area. On ADA-GEL-nBG, cells looked elongated and transformed to spindle shape.

#### 3.1.3. Cell Proliferation

**Figure 4.**
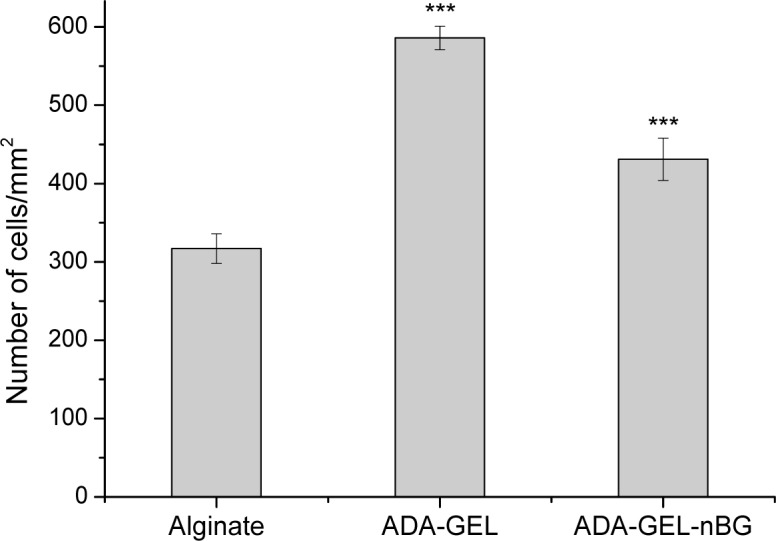
Number of cells per unit area of alginate, ADA-GEL and ADA-GEL-nBG hydrogel films after 48 h of cultivation. Alginate was used as the control material. Asterisks denote significant difference compared to alginate hydrogel, *p< 0.05, **p< 0.01 and ***p< 0.001 (Bonferroni’s post-hoc test was used).

Cell proliferation was analyzed after 48 h of cultivation and presented as number of cells per unit area of hydrogel films. As seen in Figure 4,the number of cells on ADA-GEL and ADA-GEL-nBG was significantly increased compared to alginate. The number of cells for ADA-GEL and ADA-GEL-nBG was found to be 1.8 and 1.4 folds increased compared to that for alginate, thus showing a similar pattern as mitochondrial and LDH activities.

### 3.2. In vivo Subcutaneous Implantation ofMicrocapsules

#### 3.2.1. Clinical Evaluation

All animals tolerated anesthesia and the surgical procedure well, and no rat showed signs of discomfort after surgery. A mild to moderate seroma surrounding the chambers was commonly observed, but resolved spontaneously after approximately one week. A strong inflammatory reaction could not be seen, and all animals gained weight and showed good general welfare.

#### 3.2.2. Macroscopic Appearance

At the time of explantation, two constructs of the 4 weeksADA-GEL-nBG group appeared infected and were excluded from the study. One chamber was displaced subcutaneously, so that microcapsules did not have reliable contact with the underlying muscle; this construct was also excluded.

**Figure 5.**
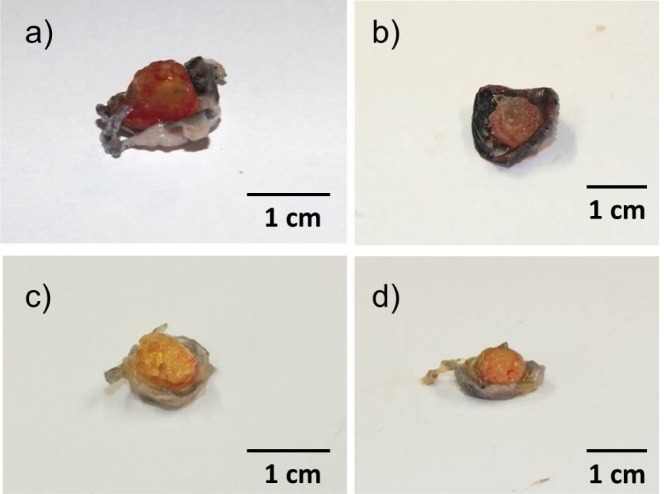
Macroscopic appearance after explantation. (**a**) ADA-GEL, 1 week; (**b**) ADA-GEL-nBG, 1 week; (**c**) ADA-GEL, 4 weeks; (**d**) ADA-GEL-nBG, 4 weeks. Microcapsules were moist and loosely adherent after 1 week (**a**,**b**) and well adherent, but often dry after 4 weeks (**c**,**d**).

After 1 week, microcapsules were soft, moist and loosely attached to the surrounding tissue (Figure 5a,b). Leakage of India ink solution from vessels was common.After four weeks, microcapsules were more adherent, and no leakage of India ink solution occurred. Microcapsules in deeper layers were firm and often macroscopically dry (Figure 5c,d).

#### 3.2.3. Microscopic Appearance

##### (1) Cellular infiltration, degradation

After one week, microcapsuleshad maintained their original shape and were surrounded by a thin layer of granulation tissue.A beginning cellular infiltration could be seen in some microcapsules.

**Figure 6.**
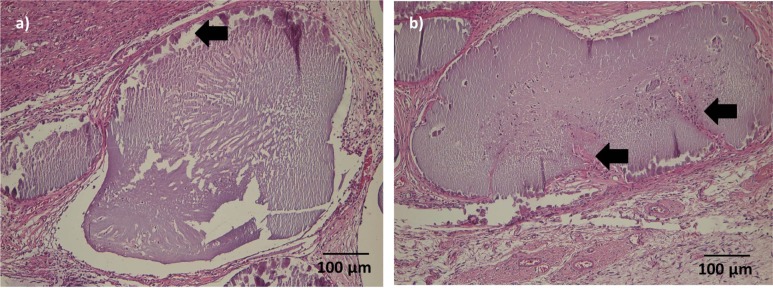
HE staining after 4 weeks; (**a**) ADA-GEL group; (**b**) ADA-GEL-nBG group; 10×. (**a**) Mild cellular infiltration had proceeded to the center of microcapsules. A thin gap between the capsule and the surrounding connective tissue was common (arrow). (**b**)Ingrowth of connective tissue into single microcapsule was visible (arrows).

After 4 weeks, the connective tissue surrounding the microcapsules was more prominent.Microcapsules directly adjacent to the muscle were of similar shape thanmicrocapsules after 1 week; cellular infiltration was mild in most cases, but had proceeded to the center of the microcapsules(Figure 6a).A thin gap between microcapsules and surrounding connective tissue/muscle could be seen in several sections. More distant microcapsules had lost their original shape and appeared homogenously eosinophilic. After 4 weeks, ingrowth of connective tissue into single microcapsule was visible (Figure 6b).

##### (2) Immune reaction

**Figure 7.**
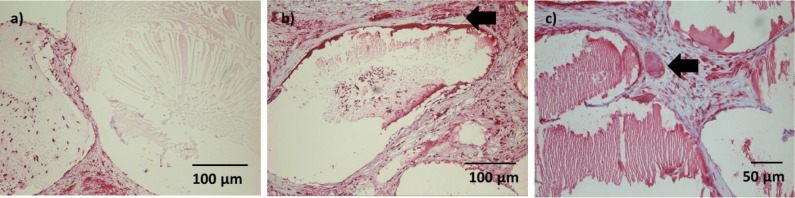
ED1 staining after (**a**) 1 week (ADA-GEL group), 10×; (**b**) 4 weeks (ADA-GEL-nBG group), 10×; **(c)** after 4 weeks (ADA-GEL-nBG group), 20×. (**a**) Grade of infiltration of microcapsules with ED1 positive cells was inconsistent; (**b**) ED1 positive cells were limited to areas directly adjacent to microcapsules after 4 weeks (arrow); (**c**) Single foreign body giant cells were detected after 4 weeks (arrow).

A moderate number of ED1 positive cells were detectable in the connective tissue after 1 week, andsome ED1 positive cells could be seen withinsingle microcapsule(Figure 7a). However, this finding was not consistent. No foreign-body giant cells were observed at this time point.

After 4 weeks, ED1 positive cells in the connective tissue were limited to the area directly adjacent to the microcapsules (Figure 7b). Infiltration of microcapsules was inconsistent. Some foreign body giant cells could be detected, with no difference between constructs with and without nBG (Figure 7c).

##### (3) Vascularization

Some Lectin positive cells were detected within the microcapsules after 1 and 4 weeks. As with ED1 positive cells, this was considered an inconsistent finding.

**Figure 8.**
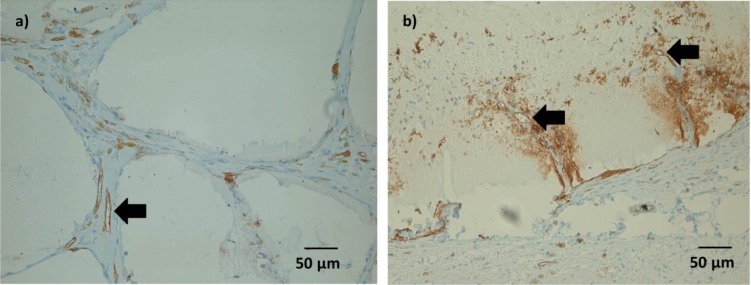
Lectin staining after four weeks (ADA-GEL-nBG group), 20×; (**a**) ED1 positive cells forming tubular structures were common in the connective tissue between the microcapsules (arrow); (**b**) formation of lectin positive tubular structures in microcapsules with connective tissue ingrowth (arrows).

After 4 weeks, immunohistochemical staining showed Lectin positive tubular structures in spaces between microcapsules, which might be a sign of ongoing vessel formation(Figure 8a). In microcapsules with connective tissue ingrowth, formation of such tubular structures could be detected also within the microcapsules (Figure 8b). However, not all of these structures were filled either with India Ink or with erythrocytes, which might question their functionality at this time point.

## 4. Discussion

Good cell viability and proliferation, as shown by increased mitochondrial and LDH activities, were found on ADA-GEL films and, albeit less pronounced, on ADA-GEL-nBG films, when compared to the control hydrogel, alginate. The increase of the mitochondrial and LDH activities of MSCs on ADA-GEL hydrogel compared to alginate can be explained by the high biodegradability and the RGD (Arg-Gly-Asp) sequence (specific cell adhesive peptide) of gelatin [25,26]. Furthermore, ADA is an oxidized product of alginate, which provides high degradability because of its low molecular weight [27]. Degradation of hydrogel enhances the mesh size and permits cell anchoring and penetration.

Mitochondrial and LDH activities of MSCs on ADA-GEL-nBG were found to be lower than on ADA-GEL. A possible explanation is a cytotoxic effect due to released ions from nBG particles [21,28]. However, this would be in contrary to results found when using rat osteoprogenitor cells, where nBG stimulated proliferation and RunX2 synthesis [20]. Osteogenic differentiation of MSCs would be another possible mechanism of changed metabolism, although it seems unlikely after only 48 h. Staining protocols such as Alizarin Red, ALP or von Kossa staining, trilineage differentiation experiments and flow cytometry of MSCs grown on ADA-GEL and ADA-GEL-nBG films should show their differentiation status and progenitor potential in future experiments.

Different morphologies were observed after 48 h of cultivation. On alginate films, cells were agglomerated and formed clusters after 48 h. Here cell-cell interactions seem to be superior to cell-material interactions, which induce the agglomeration of cells due to a lack of cell adhering biomolecules in alginate [29]. On ADA-GEL films, cells spread nicely and interacted with each other, suggesting suitable conditions for MSC growth. On ADA-GEL-nBG films, cells were elongated and transformed to spindle shape. Again, the released ions from nBG particles could have an effect on cell morphology, either by cytotoxic mechanisms or by induction of osteogenic differentiation. It is known that MSC phenotypic expression can be influenced both by soluble and by insoluble factors, such as ECM density and stiffness [30].

A mild to moderate seroma surrounding the chambers was common and resolved spontaneously after approximately one week. This reaction was most likely provoked by the teflon chamber itself, rather than the microcapsules, since this happened with several materials tested in similar experiments by our group. A strong inflammatory reaction could not be seen, despite the lack of anti-inflammatory treatment postoperatively, and all animals gained weight and showed good general welfare. This confirms the good clinical biocompatibility of this material. Leakage of India Ink after 1 week was probably due to low stability of newly formed vessels at this time point.

Degradation is a crucial factor in tissue engineering. The scaffold degradation rate should ideally match the rate of new tissue production [31]. Under good conditions, beginning bone formation can be achieved in rats as early as 2 weeks using osteoinductive and osteoconductive scaffolds [32]. In this study, a thin gap was seen between microcapsules and surrounding connective tissue in several slides from both 4-weeks-groups, and ingrowth of connective tissue into single microcapsule was visible. This would ideally match the rate of new bone formation that can be achieved in these animals.

Gelatin can be completely degraded *in vivo* by various enzymes, such as MMP-2 (Gelatinase A) or MMP-9 (Gelatinase B) [33]. Contrary to this, the *in vivo* degradation of pure alginate has been described as uncontrollable and unpredictable due to lack of natural enzymes for alginate degradation in mammals[8]. Oxidation is therefore often performed prior to crosslinking [6], as used in this study, to allow for hydrolytic *in vivo* degradation of the product. Furthermore, oxidized alginate molecules are easily be eliminated by the kidneys, in contrary to pure alginate molecules [34]. The combination of easily degradable gelatin and oxidized alginate is not expected to create any products that cannot be eliminated *in vivo* during degradation and did in fact not cause any clinical problems in this study.

The diffuse infiltration of connective tissue with ED1 positive cells seen after 1 week was probably more related to general inflammation after surgery than to the implanted material itself. After 4 weeks, ED1 positive cells were still present in a comparable number, but localized more adjacent to microcapsules, thus showing a reaction specifically induced by the implanted material. A possible explanation is the role of macrophages in degradation of the implanted matrix, such as by secreting MMP-2 and MMP-9 [35]. The low number of foreign body giant cells points to a good overall acceptance of the implanted matrix and is in accordance with previous studies evaluating the immune reaction to both gelatin [9] and alginate [4] *in vivo*. Contrary to our*in vitro* results, the addition of nBG to these materials does not seem to have any influence on their *in vivo* biocompatibility.

One of the most challenging problems in tissue engineering, especially when aiming at large, three-dimensional constructs is the need for adequate and rapid vascularization, since diffusion of nutrients and oxygen can occur only up to a distance of 200–300 μm [36]. Under ischemic conditions (low oxygen and, more importantly, low glucose), MSC viability and adenosine 5′-triphosphate (ATP) content decreases after approximately six days [37]. The presence of Lectin positive tubular structures between microcapsules in this study shows ongoing angiogenesis. However, nutrition was apparently not sufficient for deeper layers, since microcapsules situated more distant from muscular tissue and thus from nutrient vessels were often dry and had lost their original shape after 4 weeks. For large constructs, axial vascularization [38–41], the addition of growth factors to the hydrogel [42] and encapsulation of endothelial cells [43] might be a possibility to improve blood supply to the center. Furthermore, fibroblasts incorporated into alginate beads with bioactive glass have shown to produce a significant amount of VEGF [44]. VEGF increases endothelial cell survival, proliferation and migration and therefore typically mediates the first phase of capillary ingrowth [45]. Similar results might be obtained by encapsulating mesenchymal stem cells, and might also stimulate osteogenesis at the same time [4].

No differences in any of the described microscopic parameters could be seen between ADA-GEL and ADA-GEL-nBG microcapsules after subcutaneous implantation. Nano-scaled bioactive glass particles have been recently introduced in bone tissue engineering, with promising results in*in vitro* experiments. When combined with biodegradable polymers, nano-scaled particles should mimic the nano-features of the mineral phase of bone and thus increase cell adhesion and proliferation of osteogenic cells [46,47]. Several cytocompatibility studies confirmed good cell attachment and proliferation when used with different cell types [21,28,48,49]. Only a few studies have been evaluated biocompatibility of nBG particles *in vivo*[49,50] and found similar results as seen with larger bioactive glass particles [51]. The good *in vivo* biocompatibility was seen in the current study is in accordance with these results; however, our*in vitro* results suggest a cytotoxic effect of nBG on MSCs directly in contact with the material. Future studies should focus on cell survival, proliferation and differentiation potential of encapsulated cells both*in vitro* and *in vivo* as well as on determination of the ideal nBG concentration for bone tissue engineering applications.

## 5. Conclusions and Future Work

The current study shows good biocompatibility of ADA-GEL hydrogels both with and without nano-scaled bioactive glass (45S5) particles. *In vitro* experiments showed good proliferation after seeding on filmsandan increase of mitochondrial and LDH activities. However, the addition of nBG seemed to exhibit some inhibiting effect compared to pure ADA-GEL.*In vivo* implantation did not show a significant immune reaction, and ongoing degradation of the materials after four weeks was apparent, which would ideally match beginning bone formation in rats after this time. Ongoing vascularization could be seen after four weeks. No difference could be observed between pure ADA-GEL and ADA-GEL-nBG microcapsules.In conclusion, the tested materials show favorable properties for future applications in bone tissue engineering.
